# Granulocytes Affect Double-Strand Break Repair Assays in Primary Human Lymphocytes

**DOI:** 10.1371/journal.pone.0093185

**Published:** 2014-03-25

**Authors:** Sandrine Lacoste, Ravi Bhatia, Smita Bhatia, Timothy R. O'Connor

**Affiliations:** 1 Department of Cancer Biology, Beckman Research Institute, Duarte, California, United States of America; 2 Division of Hematology and Hematopoietic Cell Transplantation, City of Hope Medical Center, Duarte, California, United States of America; 3 Department of Population Sciences, City of Hope Medical Center, Duarte, California, United States of America; University of California Davis, United States of America

## Abstract

Patients who develop therapy-related myelodysplasia/acute myeloid leukemia after autologous-hematopoietic stem cell (aHCT) transplant show lower expression levels of DNA repair genes in their pre-aHCT CD34^+^ cells. To investigate whether this leads to functional differences in DNA repair abilities measurable in patients, we adapted two plasmid-based host-cell reactivation assays for use in primary lymphocytes. Prior to applying these assays to patients who underwent aHCT, we wanted first to verify whether sample preparation affected repair measurements, as patient samples were simply depleted of erythrocytes (with hetastarch) prior to freezing, which is not the classical way to prepare lymphocytes prior to DNA repair experiments (with a density gradient). We show here that lymphocytes from healthy donors freshly prepared with hetastarch show systematically a higher level of double-strand break repair as compared to when prepared with a density gradient, but that most of this difference disappears after samples were frozen. Several observations points to granulocytes as the source for this effect of sample preparation on repair: 1) removal of granulocytes makes the effect disappear, 2) DSB repair measurements for the same individual correlate to the percentage of granulocytes in the sample and 3) nucleofection in presence of granulocytes increases the level of reactive oxygen species (ROS) in neighboring lymphocytes in a dose-dependent manner (R^2^ of 0.95). These results indicate that co-purified granulocytes, possibly through the release of ROS at time of transfection, can lead to an enhanced repair in lymphocytes that obfuscates any evaluation of inter individual differences in repair as measured by host-cell reactivation. As a result, hetastarch-prepared samples are likely unsuitable for the assessment of DSB repair in primary cells with that type of assay. Granulocyte contamination that exists after a density gradient preparation, although much more limited, could have similar effects, but might be circumvented by freezing cells prior to analysis.

## Introduction

Therapy-related myelodysplasia/acute myeloid leukemia (t-MDS/AML) is a major complication of autologous-hematopoietic stem cell transplant (aHCT). Samples from patients who received aHCT for a relapsed or refractory Hodgkin's or non-Hodgkin's lymphoma have been collected for a prospective longitudinal study with the objective to identify new markers that help predict patients at risk of t-MDS/AML [1], [2]. Expression microarrays show differences between patients from the cohort that did or did not later develop t-MDS/AML [3]. Notably, a lower expression of genes implicated in DNA repair in CD34^+^ cells in peripheral blood stem cell products from patients pre-aHCT was associated with the later development of t-MDS/AML, an association that persisted in bone marrow cells at the time of diagnosis. Our ultimate goal is to verify if these differences result in functional changes in DNA repair capacities that could be more easily evaluated in a clinical setting.

Many assays exist that can be used to evaluate inter-individual differences in repair abilities. Among those, host-cell reactivation assays have the advantage to directly measure repair and can be adapted to study specific repair pathways. Moreover, the damage is generated *in vitro* prior to the introduction in the cells where the repair will be measured by the reactivation of a transgene, avoiding as much as possible concerns about cytotoxicity associated with the damage. Host-cell reactivation assays can be performed on any cell type that can be transfected, including cryopreserved primary lymphocytes [4]. Multiple population studies have used host-cell reactivation assays to evaluate DNA repair as a risk factor for several types of cancer (reviewed in [5]). We show here two host-cell reactivation assays to study independently the two pathways of double-strand bread (DSB) repair that are prevalent in non-cycling primary lymphocytes: non-homologous end-joining (NHEJ) and single-strand annealing (SSA). These assays, that we adapted for use in primary lymphocytes, can provide reproducible results in triplicates for both type of repair in 48 h starting from the cells obtained from 2.5 ml of blood, indicating that they could be applied to patient samples. However, the patients' samples we want to analyze were not prepared with this specific application in mind, but to preserve all white blood cells (WBCs) lineages for subsequent study of the progression of the disease after aHCT. To that effect, patients' blood samples were only treated with hetastarch in order to remove most of the red blood cells (RBCs) and simply frozen afterwards. But the method of choice to investigate DNA repair in peripheral blood lymphocytes is usually a density gradient that recovers mostly mononuclear cells (lymphocytes and monocytes), whereas RBCs and granulocytes sediment at the bottom of the gradient. Therefore, the main difference between the two types of preparation is related to the presence of granulocytes in addition to the lymphocytes to be studied. Granulocytes constitute 35–80% of leukocytes in the blood and are therefore the predominant cell type recovered when all WBCs are preserved, like after hetastarch aggregation. Granulocytes co-purified with peripheral blood mononuclear cells are thought to be responsible for some T-cell dysfunctions observed in samples that were not processed within 6–8 h from collection [6]–[8]. So prior to applying the new assays to patient cells, we used the blood of healthy volunteers to determine whether the type of sample preparation (density gradient or hetastarch with or without freezing), and subsequent differences in cell composition, affected DNA repair measurements.

## Materials and Methods

### Ethics statement

Use of human blood samples was approved by the City of Hope Internal Review Board: IRB protocol #98117 entitled “The Molecular Pathogenesis of Therapy-Related Myelodysplasia/Acute Myelogenous Leukemia”. Volunteers provided their written consent.

### Construction of assay plasmids

**Figure 1:**
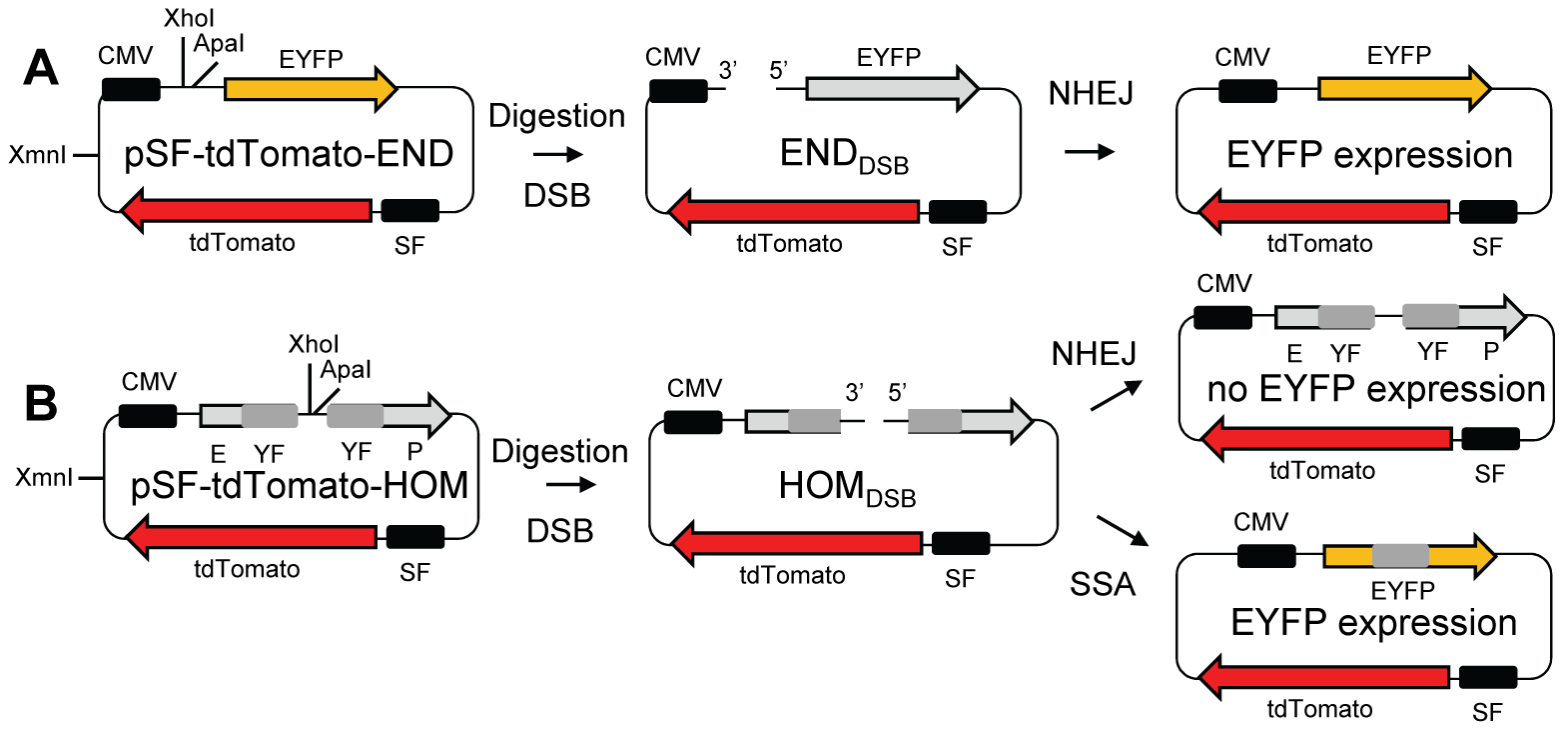
Host cell reactivation DSB repair assays. Test plasmids are digested *in vitro* with XhoI and ApaI, leading to a DSB with a 3' and a 5' overhang. Digested plasmids are transfected in cells and express tdTomato as a transfection marker and EYFP when DSB repair occurred. A functional EYFP gene is indicated in yellow. (A) pSF-tdTomato-END with a double-strand break leads to EYFP expression only after NHEJ repair restores the physical link between the gene and its CMV promoter. (B) pSF-tdTomato-HOM contains two incomplete copies of EYFP (one with the 5' and the other with the 3' part of the gene with a 355 bp overlap (grey boxes) between the two copies) resulting in a non functional gene. Repair of the DBS break in the HOM construct leads to a functional EYFP only if repair is done by SSA, any repair by NHEJ remaining undetected.

pSF-tdTomato-END (NHEJ assay) and pSF-tdTomato-HOM (SSA assay) are modified versions of pCMS-end and pCMS-hom plasmids kindly donated by R. Schiestl [9] in which the GFP transfection control was replaced with tdTomato (from ptdTomato-N1, Clontech) expressed under the spleen focus-forming virus (SF) promoter (from pHIV7-SF-RFP [10], [11]). For both modified END and HOM constructs, the DSB break in the test plasmid is generated *in vitro* by a double-digestion with ApaI and XhoI (Figure 1). Complete linearization of the plasmids is first verified by its effect on transformation efficiency (< 0.1% of undigested plasmid) but further investigation is necessary to insure complete double-digestion (XhoI and ApaI are only <75 bp apart in the constructs). For that purpose, the presence of incompatible ends is confirmed through the inability to regenerate a circular plasmid by a ligation reaction on the digested plasmids (Figure S1). Deleted plasmids pSF-tdTomato and pEYFP containing only one or the other transgene (Figure S2) were also generated to be used as single color controls during the FACS analysis.

### Cell culture materials

In all the experiments, Dulbecco's phosphate buffered saline (DPBS) is without Ca^2+^ or Mg^2+^ (Cellgro) and the culture medium is Iscove's modified Dulbecco's medium (IMDM, Gibco) with 20% heat-inactivated fetal bovine serum (FBS) (Omega Scientific).

### Cell line

GM01953 lymphoblastoid cell line (LCL) was obtained from the Coriell Cell Repository (Coriell Institute for Medical Research). Cells growing exponentially in culture medium were frozen in aliquots containing 3×10^6^ cells in 1 ml of 90% FBS with 10% DMSO (Hybri-Max, Sigma). Prior to all DNA repair experiments, cells from an aliquot were thawed and washed once in culture medium, put back in culture and allowed to recover for >7 h before nucleofection.

### Preparation of primary lymphocytes

Human blood samples (30–35 ml in heparin tubes) were obtained from 6 healthy volunteers (identifiers LYM10031 to 10036 shortened for convenience as LYM1 to LYM6) and processed ∼3 h after being drawn, except for some of the blood from LYM3 and 4 that were processed the next day when ∼29 h old. The blood was first diluted 1∶1 in DPBS containing 2% FBS. For density gradient separation, the diluted blood was transferred onto a SepMate-50 tube (Stem Cell Technologies) containing 15 ml of HISTOPAQUE-1077 (Sigma) and further processed as recommended by the tube manufacturer. For hetastarch preparation, 1 volume of HESPAN hetastarch (B Braun Medical Inc) was added to the diluted blood and mixed thoroughly. RBCs were then let to sediment for 45 min. The upper phase, containing WBCs and 5–10% of RBCs, is transferred to a new tube and washed once with DPBS and once with culture medium. For every type of preparation tested, aliquots of cells were made containing cells recovered from 2.5–3 ml of blood, either to be analyzed immediately as fresh cells or frozen aliquots for later analysis. Cell counts based on cell morphology were determined using the Hemavet 950FS (Drew Scientific).

### CD15^+^ cell depletion

Cells recovered from ∼12 ml of blood processed with hetastarch (LYM5 and 6) were subjected to an RBC lysis (see below) and then resuspended in 1.2 ml of cold buffer (DPBS pH 7.2; 0.5% FBS and 2 mM EDTA). To those cells, 280 μl of CD15 MicroBeads were added and incubated at 4°C for 15 min before wash with the buffer and passage through a 25 LS MACS separation column placed on a MACS Quadro as recommended by the manufacturer (Miltenyi Biotec). Cells that flowed through the column, mostly lymphocytes, were resuspended in culture medium. When indicated (LYM6 sample), CD15^+^ cells were recovered by rapidly flushing the column with 5 ml of cold buffer before cells were centrifuged and resuspended in culture medium.

### Freezing and thawing of blood cells

For each aliquot obtained after hetastarch or density gradient (cells recovered from 2.5–3 ml of blood), cells were resuspended in 1 ml of culture medium and 1 ml of cold freezing medium was added (60% culture medium, 20% DMSO, 20% FBS). Cell temperature was progressively decreased to −80°C in a Mr Frosty container (Nalgene) and then transferred the next day to the vapor phase of a liquid nitrogen storage tank. For thawing, 30 ml of medium containing 4,000 U/ml heparin and 62.5 μg/ml DNase were added dropwise to a rapidly thawed aliquot and cells were allowed to recover 4 h in a 37°C incubator before a 15 min spin at 200 g and resuspension in culture medium.

### RBC lysis

RBC lysis on hetastarch-prepared samples was performed by adding 9 volumes of cold ammonium chloride solution (Stem Cell technologies) to 1 volume of cells in culture medium. After 6–8 inversions, cells were centrifuged for 15 min at 200 g at 4°C before 2 washes in culture medium. Depending on the experiment, RBC lysis was performed on fresh cells or after recovery from thawing and prior to nucleofection.

### Nucleofection

Both GM01953 cells and primary lymphocytes were transfected with the P3 primary cells nucleofection kit (Lonza) using the 96-E0-115 program recommended for “high functionality” transfection of uninduced T-cells on the Amaxa Nucleofector 96-well Shuttle system. Typical transfections used 150,000–190,000 GM01953 or 125,000–200,000 primary lymphocytes (plus a variable amount of other WBCs depending on the sample preparation method). One individual aliquot of frozen GM01953 or primary lymphocytes was used for each experimental day to generate 3 measurements of each type of repair (NHEJ and SSA) plus all controls for the FACS analysis (1–3 measurements depending on the experimental day for GM01953).

### FACS analysis and DNA repair measurements

Cells transfected by nucleofection with 0.4 μg of digested plasmid DNA (END_LIN_, END_DSB_, HOM_LIN_ or HOM_DSB_) were allowed to repair DSB and express transfection (tdTomato) and repair (EYFP) reporters for 12 h before being harvested and resuspended in HBSS (compensation controls) or HBSS containing 0.3 μg/ml DAPI. Cells were then kept on ice until analysis on a BD LSRFortessa cell analyzer using FACSDiva software (version 6, BD Biosciences). Fluorescent signals were analyzed as follow: DAPI with a 355 nm UV laser (filter 450/50 nm), tdTomato with 561 nm Yellow/Green laser (filter 610/20 nm) and EYFP signal with a 488 nm blue laser (filter 525/50 nm). The PMT voltages for FSC, SSC and DAPI were kept constant for all experiments at 466 V, 237 V and 302 V respectively for lymphocytes, FSC being reduced to 350 V for GM01953 cell acquisition. The voltages for tdTomato and EYFP signals were adjusted according to the baseline established using the Cytometer Setup & Tracking beads the day of the acquisition (410 – 434 V for tdTomato and 429 – 435 V for EYFP), except for GM01953 were EYFP voltage was reduced and maintained at 370 V to compensate for a slight autofluorescence of the cells. After the acquisition, the population of lymphocytes was first selected based on their FSC and SSC values (Figure S3), and then cell doublets as well as DAPI positive cells were excluded from subsequent analysis. The only compensation necessary was set to 0.8% for tdTomato over EYFP but each sample on each experimental day had both single color controls to verify that this compensation was appropriate (Figure S2C–D and Figure S3). For each construct (END_LIN_, END_DSB_, HOM_LIN_, HOM_DSB_), the absolute recombination efficiency (ARE)  =  tdTom^+^EYFP^+^/(tdTom^+^EYFP^+^ + tdTom^+^EYFP^−^) was determined (Figure S3). The relative recombination efficiency (RRE) was then calculated for NHEJ by normalizing ARE of END_DSB_ with ARE of the END_LIN_ plasmid (represents 100% repair) (ARE_DSB_/ARE_LIN_) and for SSA by subtracting the ARE for HOM_LIN_ plasmid (ARE_DSB_ - ARE_LIN_). ARE_LIN_ represents the background of cells that show reactivation of EYFP by other means than SSA. This kind of background is almost nonexistent when analyzing normal cells such as lymphocytes or LCLs, but that is not the case for all cells types.

### Reactive oxygen species assay

The detection of reactive oxygen species (ROS) in lymphocytes 1 h after or not a mock nucleofection (cells resuspended in solution and electroporated in absence of DNA) in presence of granulocytes was performed using the CellROX Deep Red Flow Cytometry Assay kit (Molecular probes). Selection by FACS of the lymphocytes as the population of interest was performed as described above, except that dead cells were excluded using the SYTOX Blue Dead Cell Stain (405 nm Violet laser, Pacific blue filter 450/50 nm, PMT 315 V). The presence of ROS was then detected through the oxidation of the CellROX Deep Red Reagent that leads to a red fluorescent compound (640 nm Red Laser, Cy5 filter 670/30 nm, PMT 435 nm). A ROS-free negative control was generated by adding N-acetylcysteine antioxidant to lymphocytes 1 h prior to the mock transfection in order to determine the level of background Cy5 fluorescence in stained cells (gate P5 in Figure S4).

### Statistics

Two-way ANOVA was performed with GraphPad Prism 6 to evaluate the source of variation in repair (inter individual differences or sample preparation) for measurements of RRE (NHEJ and SSA) after density gradient or hetastarch. R^2^ to evaluate the correlation between RRE and percentage of granulocytes as well as between ROS level and number of granulocytes was determined using Excel.

## Results

### Plasmid constructs to monitor DSB repair

To evaluate DSB repair in primary lymphocytes, two constructs were used that allow the separate investigation of repair by NHEJ or SSA, in order to potentially identify effects specific to one pathway or the other. The original host reactivation assay system used (pCMS-end and pCMS-hom plasmids), kindly provided by Robert Schiestl [9], has the advantage of carrying a transfection control on the same molecule as the transgene measuring the repair (EYFP). As a result, the EYFP reactivation can be measured specifically in the subset of cells that were actually transfected. The original transfection control SV40-GFP in the pCMS constructs was poorly expressed in primary cells and was replaced by tdTomato expressed under the spleen focus-forming virus (SF) promoter [10], [11] to allow a balanced expression of both transgenes in hematopoietic cells and to improve discrimination between transfection control and repair signals (Figure S3). The DSB to be repaired in the plasmids is a complex break that cannot be simply repaired via ligation once in cells. This break was achieved by performing a double digestion by XhoI (generates a 3′ overhang) and ApaI (generates a 5′ overhang). Both restriction sites exist in the regions for introducing DSBs in each of the END and HOM constructs (Figure S2). The complete XhoI+ApaI double digestions were verified by analyzing religation products, which can directly demonstrate the loss of the small XhoI-ApaI fragment (Figure S1).

### Supercoiling influences transfection efficiency

Undigested supercoiled plasmids are generally used as controls for host cell reactivation assays for DSB repair to indicate 100% repair (NHEJ assay) or 0% repair (SSA assay) [9] (see calculation of recombination efficiency below). Plasmid topology can have a pronounced effect on transfection efficiency [12], [13], so we tested whether linearization in an area of the plasmid that does not affect tdTomato, EYFP nor their control sequences (XmnI in the Ampicillin resistance gene) affected the transfection efficiency of the single color pSF-tdTomato plasmid (Figure S2C). All other things being equal (cell number per transfection and nucleofection conditions), the transfection efficiency of primary lymphocytes increased with the dose of DNA used (range 0.2 – 0.8 μg) and decreased with XmnI linearization (Figure S5), on average by 63% as compared to the supercoiled plasmid. As it seems preferable that the transfection efficiency of the controls be as close as possible to the test plasmids for the host cell reactivation assays, we used XmnI-linearized plasmids (LIN) as controls instead of undigested plasmids.

### Lymphoblast and lymphocyte transfection conditions and DNA associated toxicity

**Figure 2:**
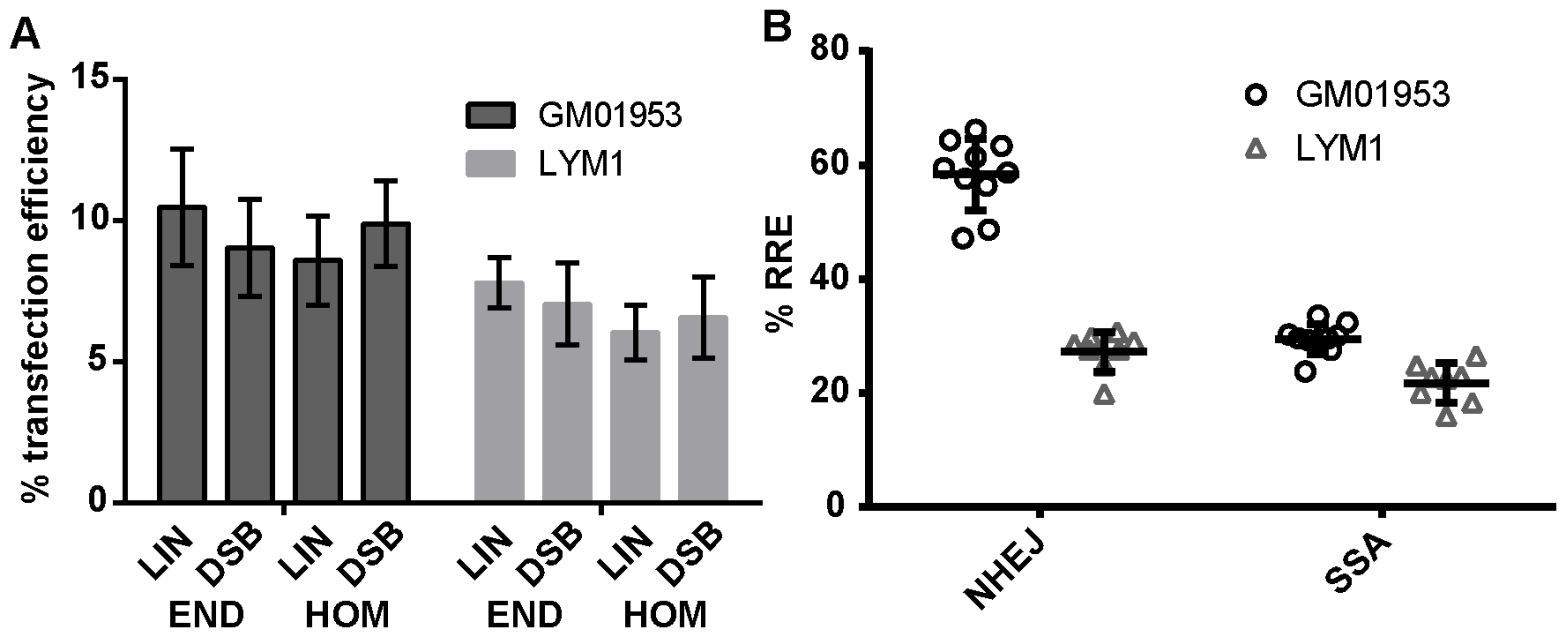
Reproducibility of the host cell reactivation assays. A) Average transfection efficiency (tdTomato^+^ cells) for each of the constructs to measure NHEJ (END_LIN_ and END_DSB_) or SSA (HOM_LIN_ and HOM_DSB_). (B) Individual measurements of the RRE by NHEJ or SSA in GM01953 lymphoblastoid cell line and in LYM1 primary lymphocytes. Data represent 8–10 measurements for each type of repair performed on 3–6 experimental days over a period of 3 months. Mean of measurements and standard deviation are indicated.

Based on the design described above, nucleofection of all digested constructs was performed on GM01953 cells (lymphoblastoid cell line), and on primary lymphocytes prepared from blood using a density gradient (LYM1). The same transfection conditions could be used for both cell types and there was no significant toxicity due to the transfection method (mock transfection in Figure S6). However, both cell types showed toxicity specifically in transfected cells in a DNA amount and time dependent manner that is consistent with the observed cell death being caused by the long term accumulation of fluorescent proteins: untransfected cells numbers remain unchanged with time, but the number of transfected cells decreases progressively with most of them being dead after 24 h (Figure S6). As DSB repair is expected to occur within the first few hours after transfection and the rest of the incubation time is mainly to achieve enough fluorescence for detection, this potential problem is avoided by properly choosing the DNA amount to be transfected (0.4 μg that gives low toxicity and high enough transfection efficiency) and the incubation time prior to analysis (12 h where fluorescent signals can be measured and cells are still alive). Using these transfection conditions, all constructs had similar transfection efficiency (Figure 2A), confirming that XmnI-linearized plasmids (LIN) are appropriate controls for DSB test plasmids. Moreover, all constructs used for the assays showed identical levels DNA-related toxicity whether they expressed either or both transgenes (data not shown), meaning that cells where repair happened (expressing both transgenes) are not more likely to die than cells expressing only the transfection tdTomato control. Therefore, no bias in the repair measurements results from DNA-related toxicity due to the experimental conditions used.

### Determination of relative recombination efficiency (RRE) in lymphoblasts and lymphocytes

We performed multiple measurements of the repair by NHEJ and SSA over a period of 3 months in both the GM01953 lymphoblastoid cell line and in primary lymphocytes LYM1. Repair is evaluated by calculating the relative recombination efficiency (RRE), which represents the proportion of cells that express EYFP^+^ among all transfected cells (tdTomato^+^) in relation to this same ratio for the control linearized plasmid (typical FACS results in LYM1 showed in Figure S3A). Figure 2B shows these individual measurements as well as the average and standard deviation, demonstrating that the host cell reactivation assays reproducibly measure repair by NHEJ and SSA in both cell types. All the measurements (8–10) were performed on at least 3 experimental days (up to 6 separate experimental days for GM01953 cells). Each experimental day started from identical frozen cell aliquots, and we did not observe more variations between experimental days (different frozen aliquots) than within repeats on the same day (several measurements from the same aliquot). The small difference in SSA repair between the GM01953 cells and LYM1 could represent different abilities between the individuals that donated their cells (RRE of 29.5%±2.7 and 21.8%±3.5, respectively). In contrast, RRE levels of NHEJ repair seems higher in lymphoblasts (RRE of 58.3%±6.3 and 27.3%±3.5, respectively). A higher repair measurement could theoretically result from a higher transfection efficiency, as more molecules transfected per cell might increase the chances of repair accordingly. However, the transfection efficiencies were similar for both cell types (Figure 2A). Moreover, no correlation could be found between RRE for NHEJ and transfection efficiencies for the individual measurements for either cell type (data not shown), thereby excluding transfection efficiency as a factor influencing the calculated RRE. Finally, we verified that RRE measurements were not altered by limited changes in transfection conditions in term of cell number (range: 100,000–250,000 lymphocytes per transfection) or DNA amount transfected (range: 0.2–0.6 μg); conditions where a low level of DNA related toxicity was observed (data not shown).

### The method of WBC purification influences subsequent DNA repair in lymphocytes

The purification of peripheral blood mononuclear cells with a density gradient is the classical way to prepare lymphocytes to measure DNA repair [4], [5], [14]. However, patients' samples in the cohort to be analyzed were harvested using hetastarch aggregation and consist of WBC-enriched samples where most RBCs were depleted prior to freezing. Density gradient and hetastarch lead to a similar yield of lymphocytes (∼1×10^6^ per ml of blood), but result in major differences in the cell composition after preparation: both RBCs and granulocytes are expected to sediment at the bottom of the tube during a density gradient isolation, whereas RBCs and granulocytes constitute the vast majority of the cells in hetastarch prepared samples. RBCs constitute ∼95% of all blood cells, so even though the hetastarch can eliminate up to 95% of them, they can still outnumber WBCs by a factor 10. RBCs hemolysate and hemoglobin have been previously reported to affect DNA repair in peripheral blood mononuclear cells [15]. Moreover, the presence of RBCs in our samples seemed to affect transfection efficiency in hetastarch-prepared lymphocytes, although in a limited manner (decrease by 25% on average when RBCs are still present). This potential issue was resolved by performing RBC lysis prior to transfection, which we verified did not affect the viability of lymphocytes nor the repair results, as long as cells had sufficient time (>3 h) to recover after thawing and prior to the RBC lysis (data not shown).

A potentially greater problem is the presence of other types of WBCs in the environment, even though flow cytometry can focus our analysis specifically on the cell type of interest (lymphocytes) by selecting specific forward and side scatter values (Figure S3A). Notably granulocytes, even in the limited number that remain after a density gradient, alter functional immunological response in T lymphocytes [7], [16]. This is thought to be caused by changes in buoyancy in activated granulocytes that happens in the long term in drawn blood which leads them to co-segregate with mononuclear cells during density gradient isolation [17]. It is therefore necessary to determine if granulocytes influence DNA repair measurements in lymphocytes.

**Figure 3:**
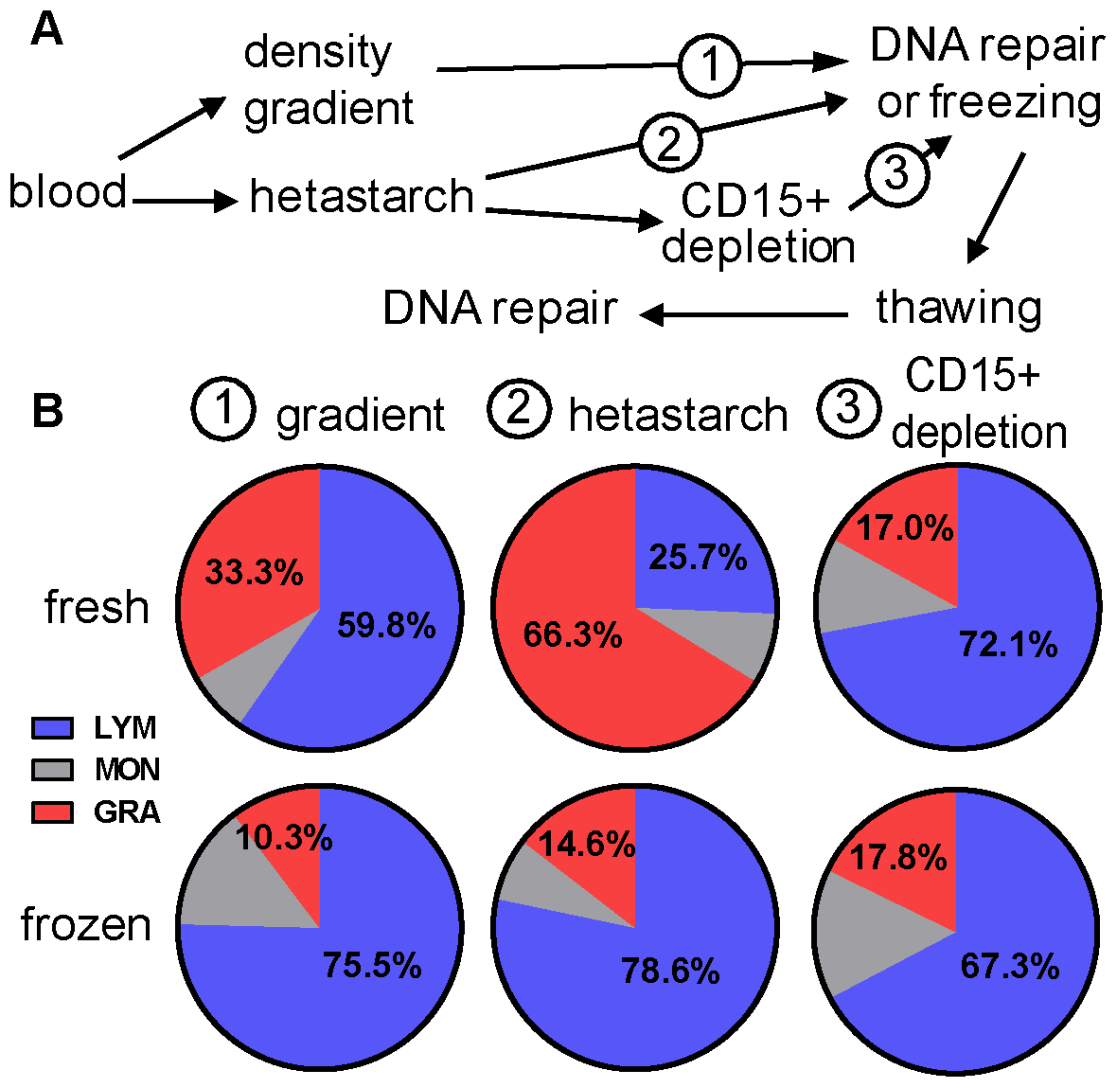
Different types of sample preparation evaluated. (A) Schematic representation of the different types of preparation protocols performed prior to repair measurements. Cells were either prepared on a density gradient (1) or incubated with hetastarch (2 and 3). When indicated for LYM5 and 6, hetastarch-preparation was followed by the depletion of CD15^+^ cells (3) (B) Cell composition of samples after purification depended on the preparation protocol that was used and whether cells were fresh or frozen. One distinctive feature of hetastarch-prepared samples is that the vast majority of cells recovered in fresh samples are granulocytes (GRA in red), whereas lymphocytes (LYM in blue) are the main cell type after density gradient preparation and/or after the cells were frozen. The last major cell type in samples are monocytes (MON).

For that purpose, lymphocytes of 5 individuals (LYM2-6) were prepared using several procedures (Figure 3A) that lead to similar yields (∼1×10^6^/ml blood) of lymphocytes (LYM) but with different levels of granulocyte (GRA) contamination in their environment (Figure 3B and Figure S7). Hetastarch-prepared samples were constituted on average of 66.3% GRA and 25.7% LYM, whereas the density gradient led to a completely inverted ratio: 33.3% GRA and 59.8% LYM. The last major cell type was monocytes (MON) that constituted around 8% of the cells in both types of preparation. Granulocytes are very sensitive to freezing and samples after thawing had similar cell composition with most of the GRA having died and mainly LYM remaining (Figure 3B).

**Figure 4:**
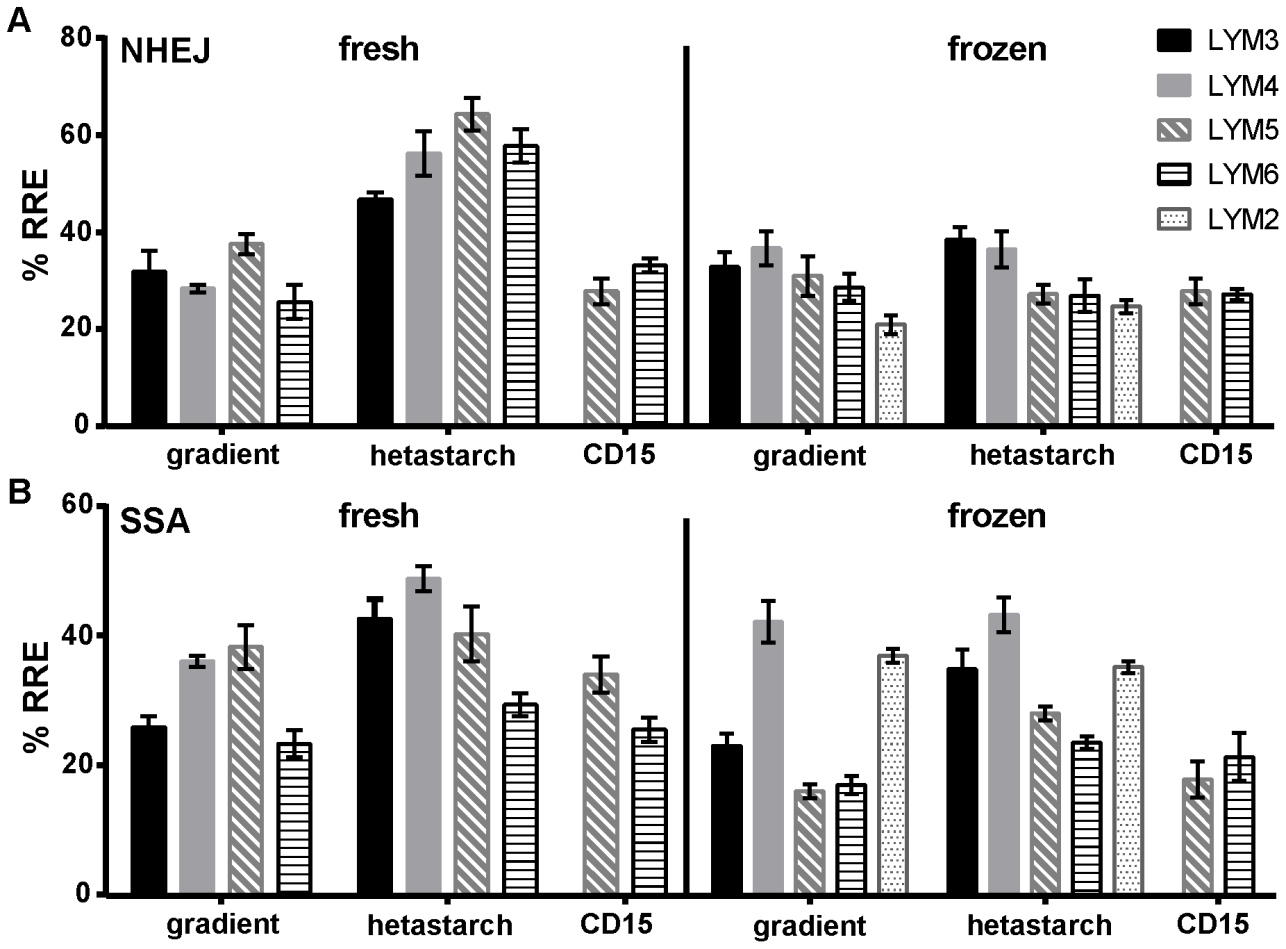
DSB repair is affected by the type of sample preparation. Repair by NHEJ (A) or SSA (B) was measured in lymphocytes from different individuals (LYM2-6). LYM2 was only analyzed after freezing. LYM3-6 were analyzed in both fresh and frozen samples, each time starting with the cells recovered from an equivalent volume of blood (∼2.5 ml). In addition, LYM 5–6 were analyzed in samples that were first hetastarch-prepared and then depleted for CD15^+^ cells. Results indicate an enhanced repair in fresh cells that were hetastarch-prepared that is strongly associated with the presence of CD15^+^ cells in the environment of the studied lymphocytes and most of that difference is gone after freezing, at least for NHEJ repair. Mean and standard deviation of 3–4 measurements on 1–2 experimental days are shown.

Repair measurements for both NHEJ and SSA in freshly prepared cells (Figure 4) were clearly higher in hetastarch samples as compared to the corresponding density gradient-prepared ones. Most of that difference had disappeared after freezing, although not completely for SSA. Statistical analysis confirmed that in fresh samples, a much more important part of the variation in measurements is associated with sample preparation (81.5% and 30.7% for NHEJ and SSA, respectively) rather than with the individual (Table 1). This effect of sample preparation was mostly lost when repair was studied after freezing, except for a small persistent effect on SSA repair (9.7% of the variation still associated with sample preparation). The difference between gradient and hetastarch-prepared samples disappeared if CD15^+^ cells were depleted (removal of 92–94% of GRA and 50–64% of MON) after hetastarch treatment and prior to the DNA repair measurement (LYM 5 and 6), indicating that the effect on repair is not related to the incubation with hetastarch, but rather linked to the presence of other cell types in the environment of the lymphocytes. Monocytes, being similar in proportion for all types of preparation, are unlikely to be the cause of the effect. There was also no correlation between DNA repair measurements and corresponding transfection efficiencies (data not shown). However, for all individuals analyzed (LYM3-6), there was a correlation between DSB repair measurements (% RRE) and the percentage of granulocytes in the corresponding sample, especially so for NHEJ (R^2^ between 0.68 and 0.82, Table 1). A comparable effect of the number of granulocytes in the mix regardless of how they were obtained indicates that the effect of sample preparation on repair is directly related to the presence of granulocytes, not to the technique used to prepare the samples *per se*.

**Table 1:** Statistical analysis of the effect of sample preparation on repair measurements.

Variable		NHEJ	SSA
individual	fresh	9.8% ****	51.6% ****
	frozen	74.7% ****	79.0% ****
sample preparation	fresh	81.5% ****	30.7% ****
(gradient or hetastarch)	frozen	0.4% ^ns^	9.7% ****
R^2^	LYM3	0.68	0.60
(% RRE *vs* % GRA)	LYM4	0.74	0.56
	LYM5	0.82	0.50
	LYM6	0.71	0.66

Variation associated with the individual or the sample preparation was investigated using ANOVA.

**** p<0.0001.

ns: not significant.

### Nucleofection affects granulocyte viability

Differences related to sample preparation described in the previous section led us to investigate possible causes of the variability observed. Delayed processing of the blood after being drawn as been associated with an increase in contaminating granulocytes and a subsequent inhibition of T cells in functional assays [7]. By definition, hetastarch preparation leads to a large number of granulocytes whether activated or not. To verify if repair was affected by that kind of phenomenon, some of the blood for LYM3 and 4 was processed much later after being drawn (29 h at room temperature instead of 3 h). After measuring repair (NHEJ and SSA) both in fresh and frozen cells, there was no difference in cell composition and/or repair associated with the waiting time before processing (data not shown).

However, an unexpected effect of our host cell reactivation assay protocol could be observed when focusing on granulocytes. Some of the CD15^+^ cells (mostly granulocytes) recovered from the column during the CD15^+^ depletion step for LYM6 (Figure S3B) were placed in culture and then submitted or not to a mock nucleofection (cells resuspended in the nucleofection solution and then electroporated in the absence of DNA). Twelve hours later, two populations of cells could be identified by FACS that differed by their forward scatter (Figure S3B): one where cells are mostly alive (>95% are DAPI negative) and one where cells were mostly dead (>90% are DAPI positive). Cells put into culture after elution from the column were still mostly in the “live cell” population, but if they were mock transfected, a massive transfer towards the “dead cell” population was observed (Figure S3B). In other words, mock nucleofection of granulocytes leads to a massive disruption of the cells' membrane integrity (DAPI can enter the cells) as well as a large decrease in cell size. This strongly suggests that the effect of nucleofection on granulocytes could be implicated in the measured differences in DNA repair observed in neighboring lymphocytes.

### CD15^+^ cells introduce oxidative stress in lymphocytes after nucleofection

**Figure 5:**
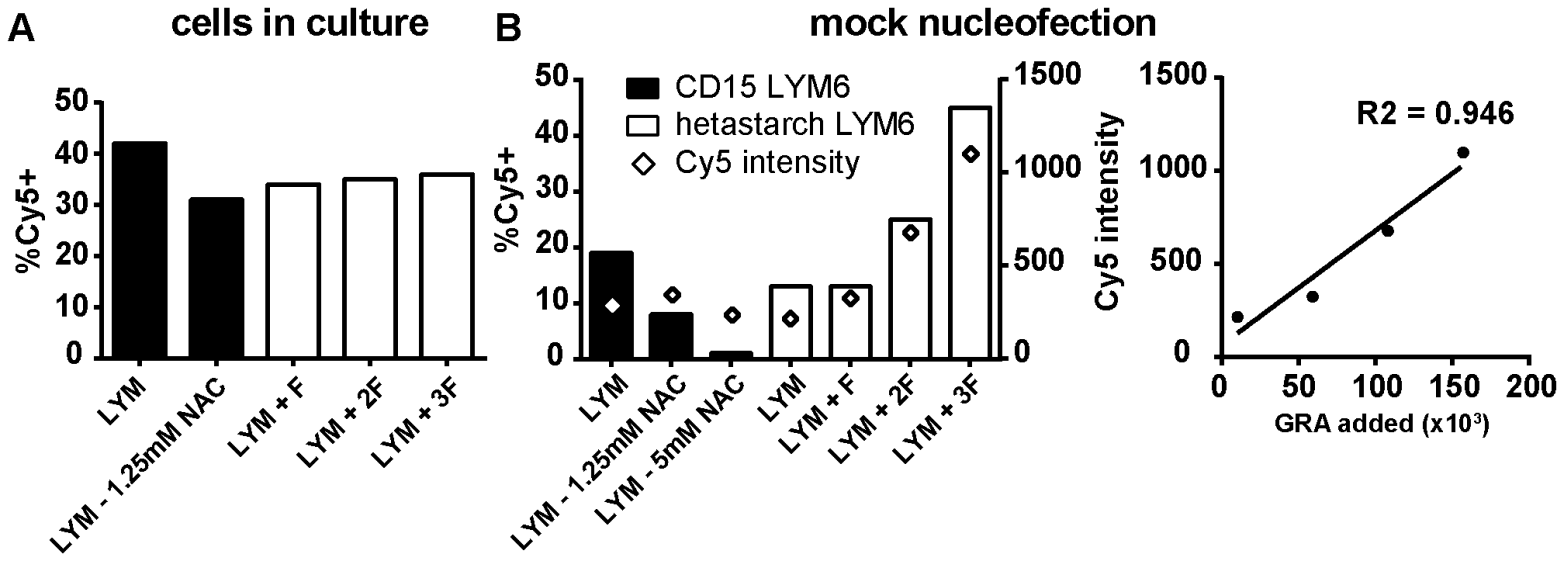
Granulocytes increase ROS in neighboring lymphocytes after nucleofection. Frozen cells samples (LYM6) that were depleted in CD15^+^ cells either before freezing (CD15 in black) of after thawing (hetastarch-prepared LYM6 in white) were analyzed for the presence of ROS after or not a mock nucleofection (cells resuspended in nucleofection solution and electroporated without DNA). Each sample contains ∼135,000 lymphocytes. When indicated, a certain fraction F (or multiples thereof) of the CD15^+^ cells recovered from the MACS depletion column were added back to the recovered lymphocytes (F≈50,000 granulocytes). (**A**) After an overnight culture, untransfected cells, regardless of how the sample was prepared, showed a subpopulation of cells with higher level of ROS measured as a % Cy5 positive cells. (**B**) When a mock transfection step was added or after treatment with the NAC antioxidant, this population of Cy5^+^ cells tended to disappear (left Y axis). However, a *de novo* formation of ROS was detected in the whole lymphocyte population one hour after nucleofection in a manner dependant of the amount (F, 2F or 3F) of CD15^+^ cells added back to the sample. As the whole population of lymphocytes shifted towards higher level of Cy5 intensity, the levels of ROS in mock transfected samples were better measured using the median Cy5 intensity in lymphocytes (right Y axis). This Cy5 median intensity was strictly correlated to the estimated number of GRA added back to the mix.

Upon necrosis of granulocytes, a massive release of granule content accompanied by the emission of a significant amount of reactive oxygen species (ROS) is expected. Such a burst of ROS into the medium could affect neighboring lymphocytes even if the nucleofection is not toxic to them. To test whether nucleofection of granulocytes affect ROS content in lymphocytes, LYM6 hetastarch-prepared cells were depleted of CD15^+^ cells immediately after thawing (see schematic diagram in Figure S4). Ten percent of the purified lymphocytes (∼135,000 cells) were used for each experimental condition. Because freezing eliminates most granulocytes, the CD15^+^ cell depletion recovered only ∼700,000 GRA and ∼300,000 MON. Different fractions F of the eluted CD15^+^ cells were then added back to the purified lymphocytes (Figure S4). F represents ∼7% of the eluate or ∼50,000 GRA. As a result, the effect of nucleofection on oxidative stress was tested on an identical number of LYM with an increasing amount of GRA in the environment (estimated cell composition of the tested conditions in Figure S4). An extra control corresponding to the lymphocytes of the same individual where CD15^+^ cells were removed prior to freezing (LYM6 - CD15 sample in Figures 3 and 4) was also used. The amount of ROS in lymphocytes placed into culture or mock nucleofected was determined using the CellROX Deep Red Reagent that leads to Cy5 fluorescence upon reaction with ROS. Cells placed into culture (Figure 5A and Figure S4 left) show two populations of cells (2 peaks based on Cy5 intensity), with 30–40% of the cells in all samples being in the Cy5 positive category, including for the CD15 control and in all other samples regardless of the amount of granulocytes added back (Figure S4 left). This Cy5 positive population of cells had a stable mean fluorescence and disappeared in presence of the antioxidant N-acetylcystein (NAC) (measured by a decrease in the % Cy5^+^ positive cells). In samples that were mock transfected (Figure 5B and Figure S4 right), the population of Cy5^+^ cells also seemed to disappear, although it is unclear why the mock transfection would have such an effect. This observation could be related to a change in culture medium associated with that step and/or an antioxidant effect of the proprietary nucleofection solution (Lonza). Either way, there was clearly a *de novo* formation of ROS 1 h after mock nucleofection of cells in a granulocyte amount-dependent manner (Figure 5B). This ROS formation is illustrated by a general shift of the whole lymphocyte population towards higher level of Cy5 fluorescence (Figure S4), which is best measured using the mean Cy5 intensity in the cells. In the hetastarch-prepared LYM6, this mean value of median Cy5 intensity was strictly correlated to the estimated number of granulocytes in the mix at the time of nucleofection (Figure 5B). Therefore, electroporation of lymphocytes in presence of granulocytes leads to a related exposure to ROS just as lymphocytes are starting to repair the transfected constructs. We could not verify if this resulted in measurable differences in repair because only a limited number of cells were available.

## Discussion

The host-cell reactivation assays modified for primary lymphocytes that we describe here successfully measure DSB repair by NHEJ and SSA starting from a limited number of cells in both lymphoblasts and density gradient-prepared primary lymphocytes. Reproducible measurements can consistently be achieved when repeating measurements from the same preparation (*i.e.* starting from different frozen aliquots), but different preparation protocols starting from the same blood sample can lead to different repair measurements for the same individual. Notably, fresh hetastarch-prepared cells (that contain all WBC types) show systematically higher levels of repair in lymphocytes compared to their density gradient-prepared counterparts, although the analysis is focused in both cases on the same cell type. The single most likely factor responsible for this effect is the presence of granulocytes at the time of transfection in the vicinity of the lymphocytes in which the repair is measured. Granulocytes carry granules in their cytoplasm that contain toxic materials that help eliminate unwanted microorganisms after phagocytosis. They are short-lived in the bloodstream and activated granulocytes are thought to be responsible for the suppression of T-cell function that is observed in advanced cancer patients [17]. Granulocytes are supposed to be eliminated when processing blood on a density gradient because their density leads them to pellet at the bottom of the tube, but this was not our experience in the present work where granulocytes consistently represented around one third of the cells recovered after this type of preparation. Others have reported variable results in cell composition in clinical samples [8] and it is unclear if our results are truly atypical for what can be achieved with density gradients and/or SepMate tubes or whether it reflects the fact that, in most case, the tools are not available to researchers to easily identify the cell types among recovered WBCs, and therefore the assumption is that these cells are only mononuclear cells when they are in fact not. Although not granulocyte-free, density gradient prepared samples still allowed to measure repair in presence of a comparatively low number of granulocytes, especially after freezing. Granulocyte sensitivity to freezing is the likely explanation for the reduced effect of sample preparation in frozen samples. One way granulocytes could affect lymphocytes is by releasing their granule content in the culture medium, resulting in an oxidative stress in lymphocytes and we showed that ROS are indeed increased after nucleofection in lymphocytes, in a manner dependent on the number of granulocytes in their environment. It is important to note that the effect of granulocytes on repair seems closely associated with the release of ROS species at the transfection step and therefore might not affect DNA repair analyses that do not include such a step, such as the follow-up of DNA repair foci. Although we did not have enough cells to verify that the increase in granulocyte-related ROS resulted in actual differences in DSB repair, we found a good correlation between the percentage of granulocytes measured and the repair for the corresponding samples of any given individual. The exposure to low doses (1–70cGy) of ionizing radiation (IR) has been shown in multiple models, including in primary lymphocytes, to improve the subsequent repair of comparatively higher doses (5–7Gy) of IR [18]–[20]. This adaptive response is associated with changes in gene expression, notably in DNA damage response genes [21]–[24]. Lymphocytes transfected in presence of granulocytes are exposed simultaneously to ROS (granulocyte related) and DSB (from the transfected plasmids) and it is conceivable that they could respond to such signals in a manner similar to an exposure to IR. In the present system, different levels of oxidative stress seem to influence directly DSB repair in an unrelated plasmid system in the same cells. A dose-dependent effect of ROS indicates that oxidative stress might be a signal leading to an enhanced DSB repair response in primary lymphocytes.

The results obtained for frozen density gradient-prepared samples are likely the closest to a repair not influenced by the presence of granulocytes and allow us to look at inter individual differences in SSA and NHEJ repair. SSA in lymphoblasts (RRE = 29.5%) was within the range measured for other individual's primary lymphocytes (RRE between 16.2% and 42.4%). However, NHEJ in lymphoblasts (RRE = 58.4%) seemed radically more efficient compared to the repair in lymphocytes (RRE between 21.3% and 36.7%). Although one cannot exclude that the individual whose cells were used to generate the GM01953 cells line had higher repair capacities, the high repair could reflect a better ability for NHEJ in cycling and/or transformed cells where the risk of DSB is expected to be higher than in G_o_ lymphocytes [25]. It could also be reflect the fact that measurements were performed in different cells types (T-cells for LYM, B cells for GM01953).

## Conclusions

The protocol used to prepare blood cells for phenotypic DSB repair assays in peripheral primary lymphocytes can alter the results to the point where inter individual differences can be completely obfuscated. This effect is likely caused by the variable presence of granulocytes in the sample and precludes the use of alternatives to density gradient to recover lymphocytes. As a consequence, great care should be taken to minimize the level of granulocyte contamination if DNA repair by host cell reactivation assay is the purpose of any cell preparation.

## Supporting Information

Figure S1 **Verification of the complete XhoI + ApaI double digestion prior to transfection.** (A) The complete double digestion can be verified by studying the ability to recircularize the plasmids or not after linearization. The theoretical products are shown here for the pSF-tdTomato-END plasmid but would be the same when verifying the pSF-tdTomato-HOM double digestion, except that a SalI site is used instead of EcoRI for the secondary digestion. Plasmids digested with a single enzyme (ApaI) can be recircularized as a single plasmid (*) or a dimer (*Δ), whereas double-digested plasmid (ApaI + XhoI) can only generate linear dimers (Δ) or trimers after ligation. The removal of the excised XhoI - ApaI fragment is further verified by a secondary digestion of the ligation products cutting inside of this fragment. Recircularized plasmids (*) can be linearized again with this secondary digestion, whereas the ApaI + XhoI double-digested DNA have lost the target restriction site and are not affected. Linear dimers are obtained with the secondary EcoRI digestion of the recircularized dimers (*Δ) when the plasmids were religated in head-to-head orientation. (B) The results of the religation experiment can be analyzed on an agarose gel where the different ligation products have different migration patterns. The secondary digestion is EcoRI for END or SalI for HOM.
(TIF)

Figure S2 **Map of plasmids used in the host cell reactivation assays.** (A) pSF-tdTomato-END used to measure NHEJ. (B) pSF-tdTomato-HOM used to measure SSA. (C) and (D) Deleted plasmids expressing a single fluorescent protein used as compensation controls for the FACS analysis.
(TIF)

Figure S3 **Typical FACS data.** (A) Lymphocytes (P1) in red are the population of interest for the DNA repair assays (in this example: frozen hetastarch-prepared LYM5). DAPI staining is used to eliminate dead cells (in blue) from the analysis and to delineate the quadrants separating negative and positive populations. Control single color plasmids are used to verify that compensation is appropriate. For each digested construct (END_LIN_, END_DSB_, HOM_LIN_, HOM_DSB_), the absolute recombination efficiency (ARE  =  Q2/(Q1+Q2)) is determined. The relative recombination efficiency (RRE) is then calculated for NHEJ by normalizing data for END_DSB_ with ARE of the END_LIN_ plasmid (represents 100% repair) (ARE_DSB_/ARE_LIN_) and for SSA by subtracting the ARE for HOM_LIN_ plasmid (represents no repair) (ARE_DSB_ – ARE_LIN_). (B) Effect of a mock nucleofection on fresh granulocytes. After elution from the CD15^+^ depletion column, LYM6 granulocytes were put back into culture and mock nucleofected (electroporated without DNA) or not in conditions identical to those used for the DNA repair assays. In a FACS analysis, CD15^+^ cells (mostly granulocytes) present as two populations that differ mainly by their forward scatter: P1 (in red) is mostly live cells (>95% are DAPI negative) and P5 (in blue) is mostly dead cells (>90% are DAPI positive). Untransfected cells are mostly in the P1 population, whereas mock transfected cells are overwhelmingly in the P5 population, indicating massive level of granulocyte cell death upon mock nucleofection.
(TIF)

Figure S4 **ROS measured in LYM6.** Samples were depleted of CD15^+^ cells in freshly prepared cells (A) or after thawing (B). For both types of preparation (from the same donor LYM6), cells in culture show a subpopulation of cells that have a Cy5 signal above background measured as the % Cy5^+^ cells (P5 gate). This specific population tends to disappear in presence of an antioxidant (NAC) and/or after mock nucleofection. However, nucleofection in presence of increasing number of CD15^+^ cells added back in the cell mix leads to a dose-dependent general shift of the lymphocyte population towards higher level of ROS as measured by a change in the median Cy5 value in the whole population. The estimated cell composition of the tested samples is shown (bottom right).
(TIF)

Figure S5 **Effect of linearization on transfection efficiency.** For all DNA amount tested, the transfection efficiency in primary lymphocytes LYM1 of XmnI-linearized pSF-tdTomato is decreased compared to the same amount of supercoiled undigested plasmid.
(TIF)

Figure S6 **Time-dependent toxicity associated with DNA after nucleofection.** (A) GM01953 LCLs and (B) LYM1 primary lymphocytes were transfected with the same amount of XmnI-linearized END control (END_LIN_) that expresses both tdTomato and EYFP constitutively. Live (DAPI negative) cells in the populations of interest are shown in red. For both cell types, the population of transfected cells (Q1+Q2+Q4) decreased with time after transfection (12 h, 16 h or 24 h), whereas mock or untransfected cells (Q3) were not affected, indicating toxicity specifically associated with the expression of the transgenes and not the transfection protocol *per se*.
(TIF)

Figure S7 **Cell composition of analyzed samples.** The proportion of lymphocytes (in blue) and granulocytes (in red), as determined with a Hemavet 950FS, are indicated for each sample analyzed for DNA repair.
(TIF)
