# Supplementary figures and images for: Granulocytes Affect Double-Strand Break Repair Assays in Primary Human Lymphocytes
Source: PLoS One. 2014 Mar 25;9(3):e93185. doi: 10.1371/journal.pone.0093185 (PMC3965556; doi:10.1371/journal.pone.0093185)

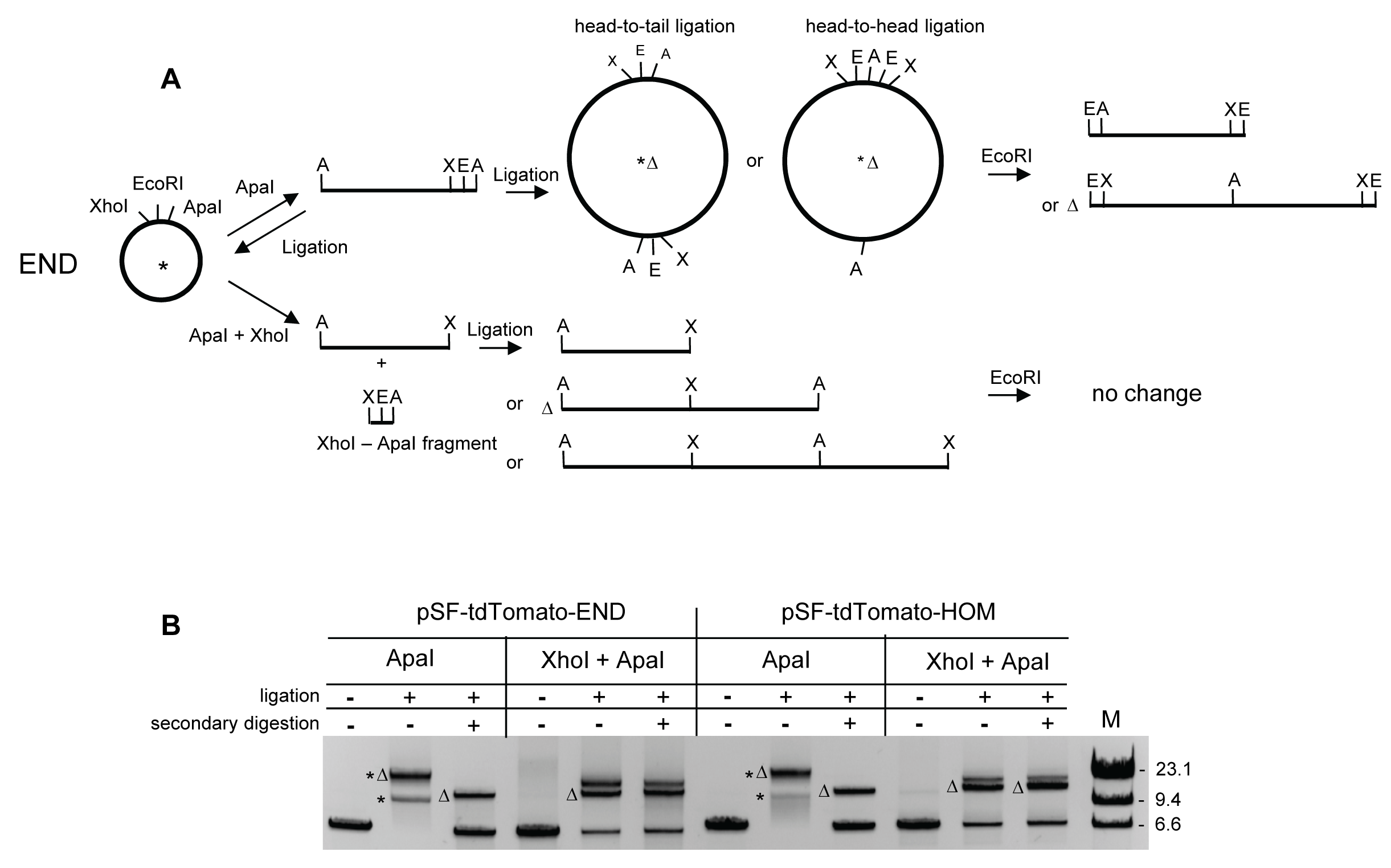

Supplement: Figure S1 — Verification of the complete XhoI + ApaI double digestion prior to transfection. (A) The complete double digestion can be verified by studying the ability to recircularize the plasmids or not after linearization. The theoretical products are shown here for the pSF-tdTomato-END plasmid but would be the same when verifying the pSF-tdTomato-HOM double digestion, except that a SalI site is used instead of EcoRI for the secondary digestion. Plasmids digested with a single enzyme (ApaI) can be recircularized as a single plasmid (*) or a dimer (*Δ), whereas double-digested plasmid (ApaI + XhoI) can only generate linear dimers (Δ) or trimers after ligation. The removal of the excised XhoI - ApaI fragment is further verified by a secondary digestion of the ligation products cutting inside of this fragment. Recircularized plasmids (*) can be linearized again with this secondary digestion, whereas the ApaI + XhoI double-digested DNA have lost the target restriction site and are not affected. Linear dimers are obtained with the secondary EcoRI digestion of the recircularized dimers (*Δ) when the plasmids were religated in head-to-head orientation. (B) The results of the religation experiment can be analyzed on an agarose gel where the different ligation products have different migration patterns. The secondary digestion is EcoRI for END or SalI for HOM. (TIF) [file pone.0093185.s001.tif]

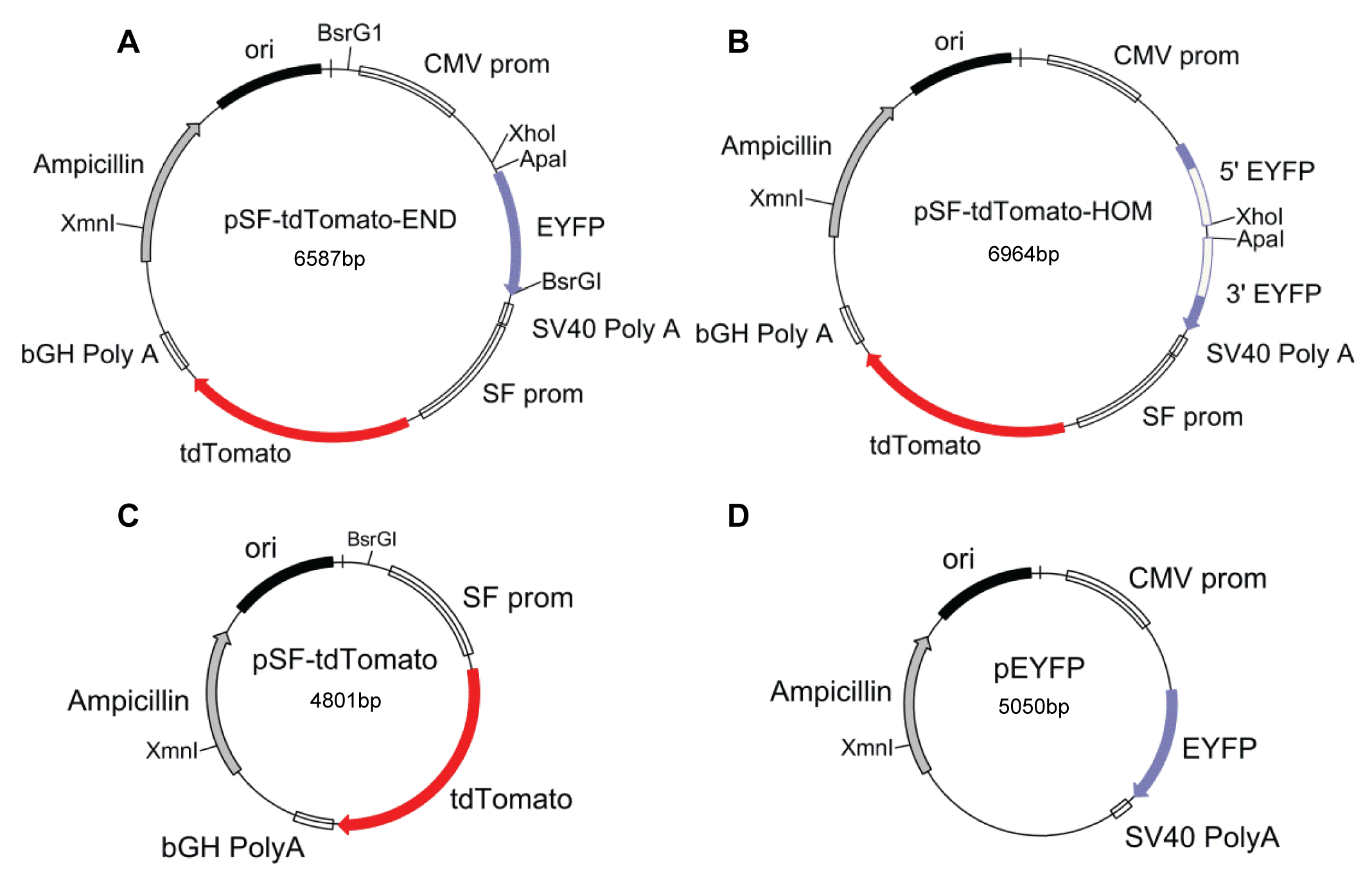

Supplement: Figure S2 — Map of plasmids used in the host cell reactivation assays. (A) pSF-tdTomato-END used to measure NHEJ. (B) pSF-tdTomato-HOM used to measure SSA. (C) and (D) Deleted plasmids expressing a single fluorescent protein used as compensation controls for the FACS analysis. (TIF) [file pone.0093185.s002.tif]

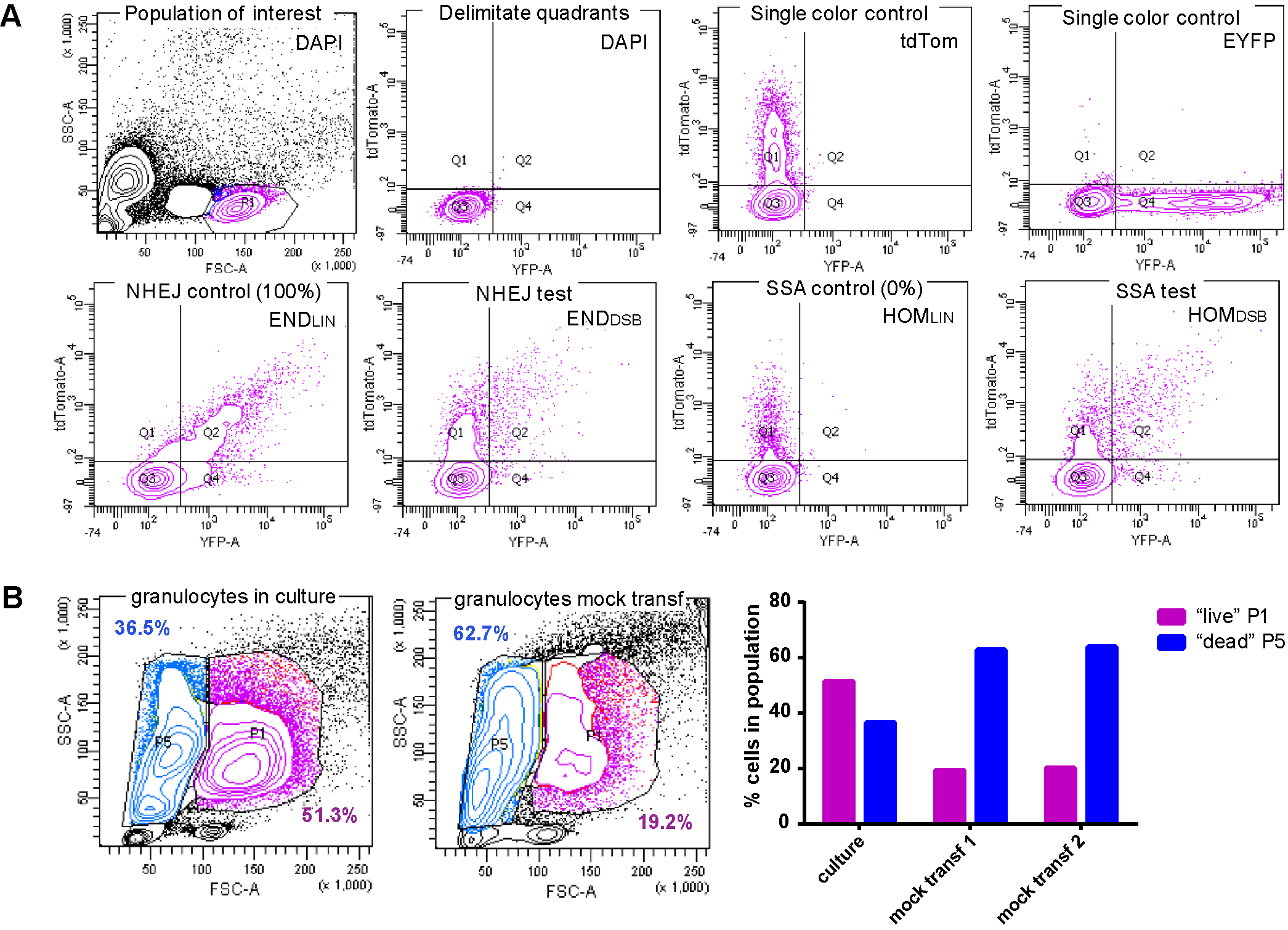

Supplement: Figure S3 — Typical FACS data. (A) Lymphocytes (P1) in red are the population of interest for the DNA repair assays (in this example: frozen hetastarch-prepared LYM5). DAPI staining is used to eliminate dead cells (in blue) from the analysis and to delineate the quadrants separating negative and positive populations. Control single color plasmids are used to verify that compensation is appropriate. For each digested construct (ENDLIN, ENDDSB, HOMLIN, HOMDSB), the absolute recombination efficiency (ARE = Q2/(Q1+Q2)) is determined. The relative recombination efficiency (RRE) is then calculated for NHEJ by normalizing data for ENDDSB with ARE of the ENDLIN plasmid (represents 100% repair) (AREDSB/ARELIN) and for SSA by subtracting the ARE for HOMLIN plasmid (represents no repair) (AREDSB – ARELIN). (B) Effect of a mock nucleofection on fresh granulocytes. After elution from the CD15+ depletion column, LYM6 granulocytes were put back into culture and mock nucleofected (electroporated without DNA) or not in conditions identical to those used for the DNA repair assays. In a FACS analysis, CD15+ cells (mostly granulocytes) present as two populations that differ mainly by their forward scatter: P1 (in red) is mostly live cells (>95% are DAPI negative) and P5 (in blue) is mostly dead cells (>90% are DAPI positive). Untransfected cells are mostly in the P1 population, whereas mock transfected cells are overwhelmingly in the P5 population, indicating massive level of granulocyte cell death upon mock nucleofection. (TIF) [file pone.0093185.s003.tif]

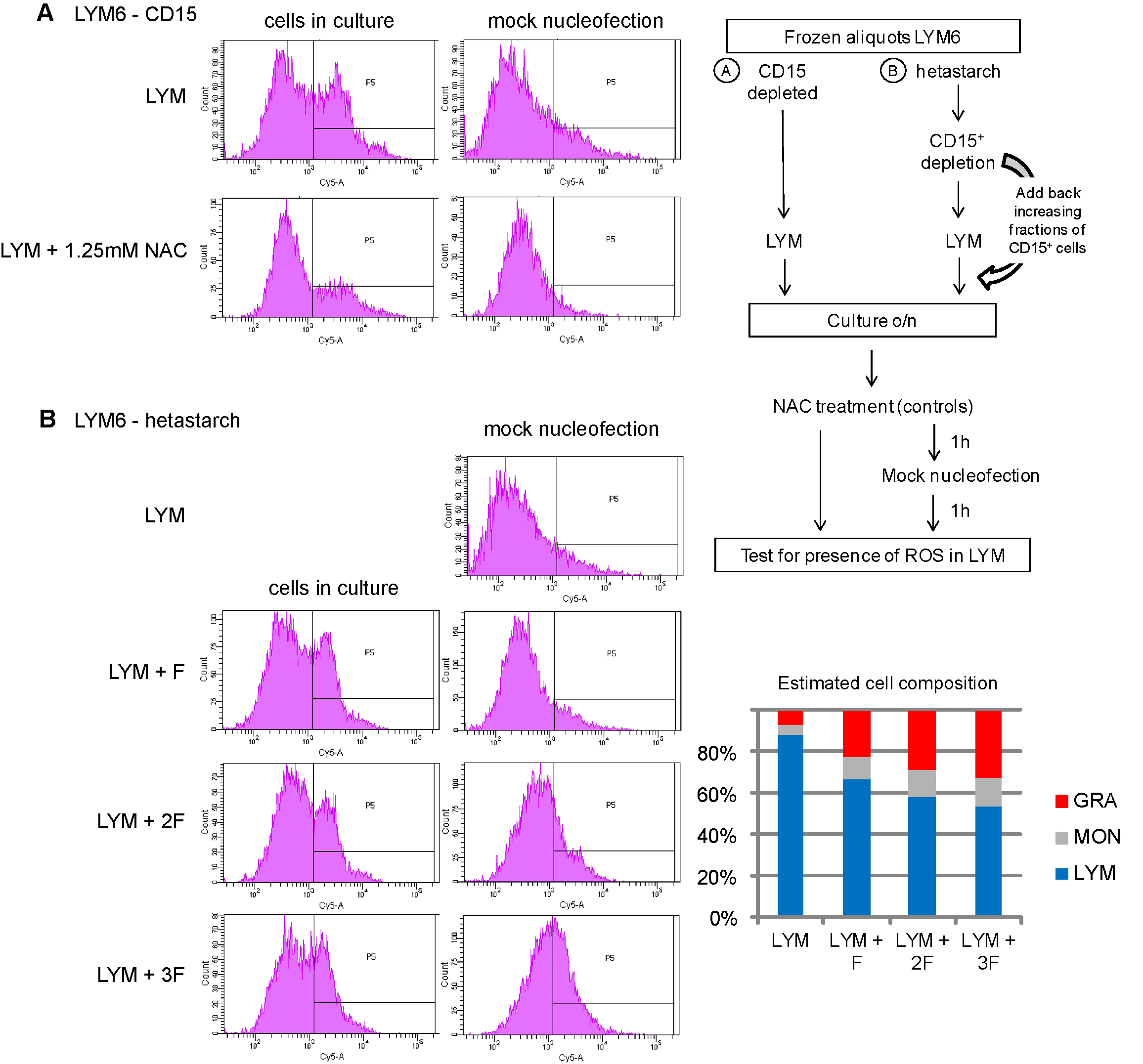

Supplement: Figure S4 — ROS measured in LYM6. Samples were depleted of CD15+ cells in freshly prepared cells (A) or after thawing (B). For both types of preparation (from the same donor LYM6), cells in culture show a subpopulation of cells that have a Cy5 signal above background measured as the % Cy5+ cells (P5 gate). This specific population tends to disappear in presence of an antioxidant (NAC) and/or after mock nucleofection. However, nucleofection in presence of increasing number of CD15+ cells added back in the cell mix leads to a dose-dependent general shift of the lymphocyte population towards higher level of ROS as measured by a change in the median Cy5 value in the whole population. The estimated cell composition of the tested samples is shown (bottom right). (TIF) [file pone.0093185.s004.tif]

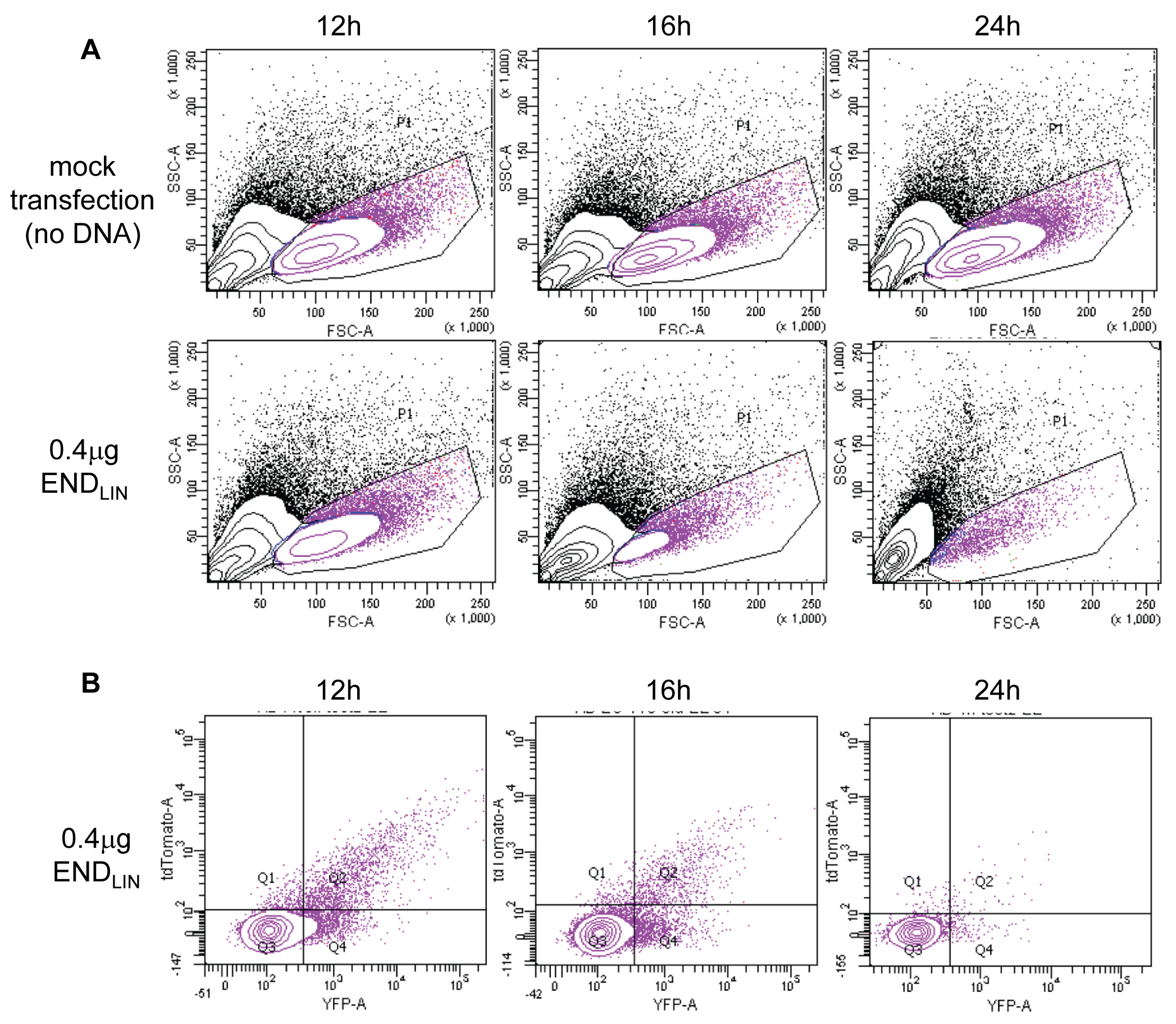

Supplement: Figure S6 — Time-dependent toxicity associated with DNA after nucleofection. (A) GM01953 LCLs and (B) LYM1 primary lymphocytes were transfected with the same amount of XmnI-linearized END control (ENDLIN) that expresses both tdTomato and EYFP constitutively. Live (DAPI negative) cells in the populations of interest are shown in red. For both cell types, the population of transfected cells (Q1+Q2+Q4) decreased with time after transfection (12 h, 16 h or 24 h), whereas mock or untransfected cells (Q3) were not affected, indicating toxicity specifically associated with the expression of the transgenes and not the transfection protocol per se. (TIF) [file pone.0093185.s006.tif]

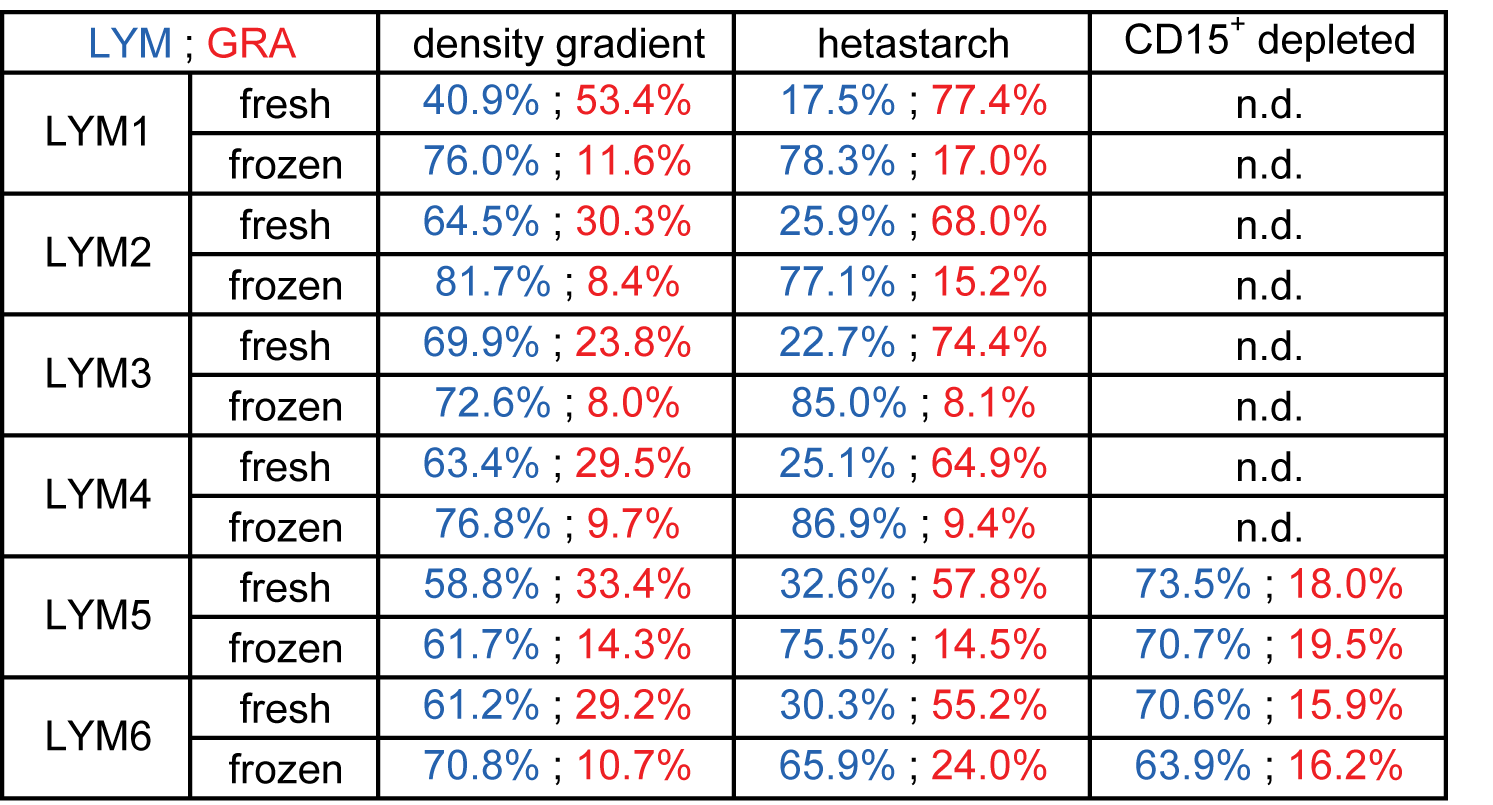

Supplement: Figure S7 — Cell composition of analyzed samples. The proportion of lymphocytes (in blue) and granulocytes (in red), as determined with a Hemavet 950FS, are indicated for each sample analyzed for DNA repair. (TIF) [file pone.0093185.s007.tif]
